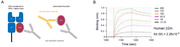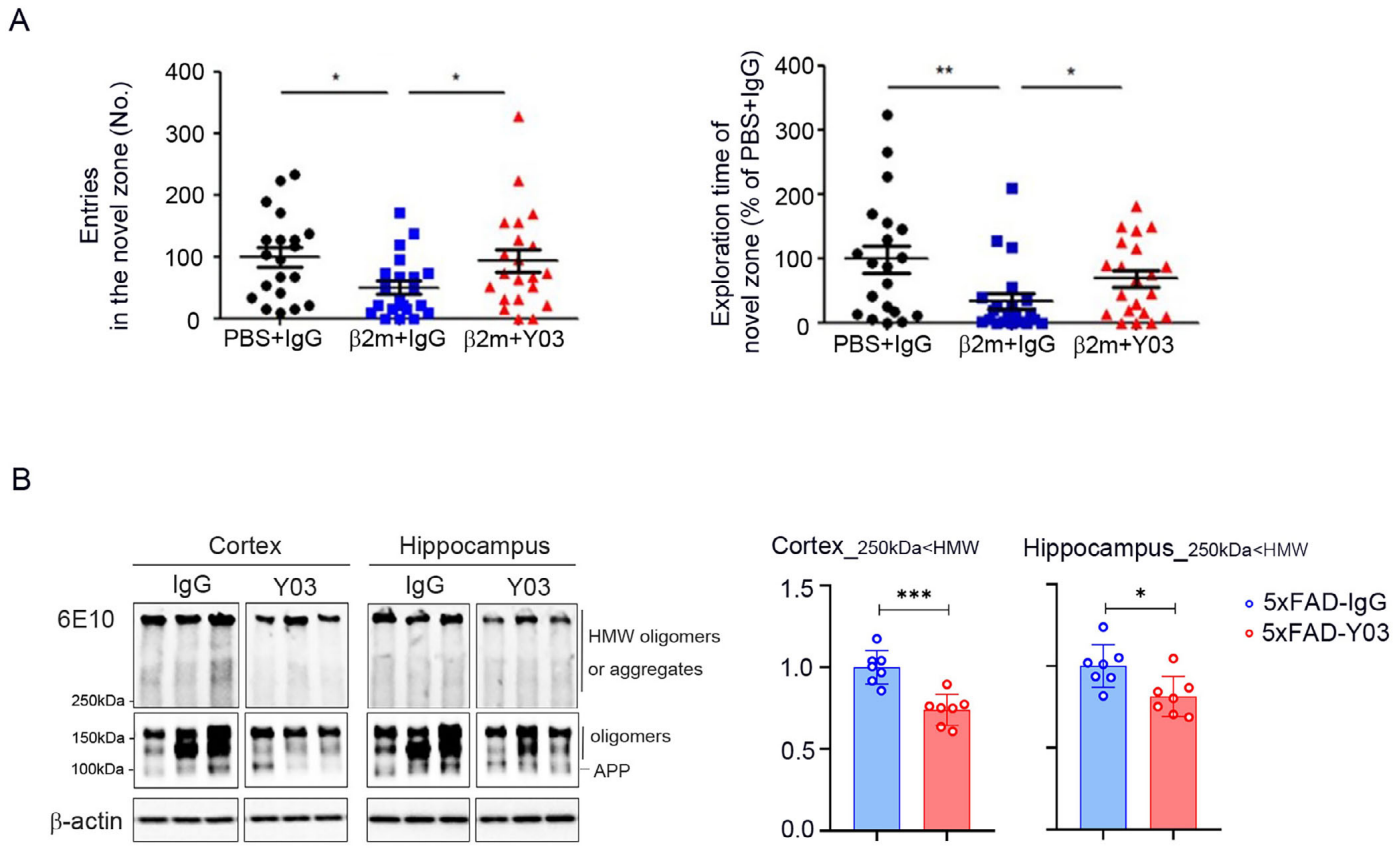# Selective targeting free beta‐2 microglobulin as a promising therapeutic strategy for Alzheimer’s Disease

**DOI:** 10.1002/alz.088124

**Published:** 2025-01-03

**Authors:** Mi‐Hyang Cho, Min‐Seok Kim, Seung‐Hwan Han, Yeon‐Seon Mun, Ha‐Lim Song, Na‐Young Kim, Jung‐Su Shin, Dong‐Hou Kim, Seung‐Yong Yoon

**Affiliations:** ^1^ ADEL Institute of Science & Technology (AIST), ADEL, Inc., Seoul Korea, Republic of (South); ^2^ Asan Medical Center, University of Ulsan College of Medicine, Seoul Korea, Republic of (South)

## Abstract

**Background:**

Beta‐2 microglobulin (β2m) is a component of the major histocompatibility complex class I (MHC‐I) playing a crucial role in the immune system on cell surface, but it can be separated from the MHC‐I and exist in biological fluid independently. Numerous reports have shown that β2m is a systemic pro‐aging factor impairing cognitive function, and that it is increased in the blood and CSF of patients with Alzheimer’s disease (AD). While β2m in the body fluid has been recognized as a potential factor in AD and aging, the development of therapeutic agents, especially those directly targeting β2m using antibodies, may face challenges. As β2m is a protein normally expressed in most nucleated cells as heterodimer bound to MHC‐I, targeting β2m may indeed carry a risk of compromising immune responses and leading to side effects by inhibiting MHC‐I.

**Method:**

To develop monoclonal antibody against free β2m, not complexed to MHC‐I, the hybridoma clones were obtained from mice injected with peptides containing epitopes targeting the adjacent region where β2m binds to heavy chain, another subunit of MHC‐I. One of these antibody clones, termed ADEL‐Y03, was selected by characterizing its binding affinity and specificity for free β2m, and mass‐produced for in vivo efficacy study against AD.

**Result:**

The ADEL‐Y03 exhibit high affinity and specificity for free β2m, but not to β2m complexed with MHC‐ I. The in vivo activity of ADEL‐Y03 in blocking free β2m was validated by recovering β2m‐induced cognitive deficit in wild type mice. In addition, intraperitoneal injection of ADEL‐Y03 reduced free β2m levels in the brain and plasma, leading to a decrease of beta‐amyloid and inflammation, hallmarks of AD pathology, in 5XFAD mice brain. In vitro study demonstrates the role of free β2m in AD pathology, and these effects were ameliorated by ADEL‐Y03.

**Conclusion:**

Specific targeting free β2m using ADEL‐Y03 antibody may be a promising approach to developing new therapies for AD without deleterious effects such as downregulating immune function of MHC‐I.